# Pre-hospital resuscitative endovascular balloon occlusion of the aorta

**DOI:** 10.1186/1757-7241-22-S1-P19

**Published:** 2014-07-07

**Authors:** Nina Gjerde Andersen, Marius Rehn, Marianne Oropeza-Moe, Nils Petter Oveland

**Affiliations:** 1 Department of Research and Development, Norwegian Air Ambulance Foundation, Droebak, Norway; 2 Department of Anesthesiology and Intensive Care, Akershus University Hospital, Oslo, Norway; 3 Department of Health Studies, Network for Medical Sciences, University of Stavanger, Stavanger, Norway; 4 Department of Production Animal Sciences, Section of Small Ruminant Research, Norwegian School of Veterinary Science, Norway; 5 Department of Anesthesiology and Intensive Care, Stavanger University Hospital, Stavanger, Norway

## Background

Internal bleeding from pelvic fractures is difficult to stop with external compression and necessitates other resuscitation techniques such as endovascular balloon occlusion of the aorta (REBOA). This minimally invasive procedure stops the bleeding by inflating a balloon in the aorta, and has been used successfully in emergency departments and operations theaters. If REBOA also is feasible in the pre-hospital environment is unknown.

## Study objectives

To present two supplementary training methods for REBOA; A gelatin model for repetitive exercise of technical skills and a porcine model for “live-tissue” training and evaluation of the hemodynamic effects of the aortic occlusion.

## Method

A gelatin-mannequin, containing water filled bicycle tubes to mimic the aorta and femoral arteries, was casted. A doctor-paramedic crew performed the REBOA procedure three times in a realistic pre-hospital environment. The time from arrival to balloon-inflation and skin contact to balloon-inflation was recorded.

A porcine model (blood volume 3640 mL) was bled (200 mL of blood withdrawn) in eight consecutive steps to a total blood loss of 44%. The balloon was inserted through the femoral artery and into the aorta using an ultrasound guided Seldinger-technique, and inflated and deflated after each bleeding step. The systolic (SBP) and diastolic (DBP) blood pressures were measured.

## Results

**Figure 1:**
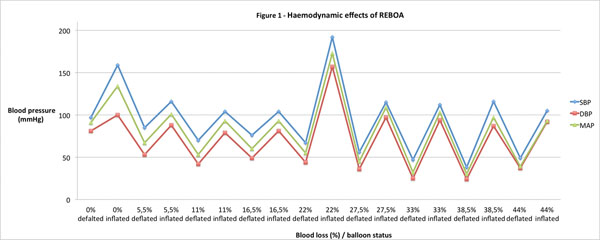
Hemodynamic effect of REBOA. The peaks show the increase in blood pressure after inflation of the balloon at different levels of blood loss. SBP: systolic blood pressure; DPB: diastolic blood pressure; MAP: mean arterial pressure.

The mean time from arrival to inflation of the balloon was 4 minutes and 19 seconds (SD ± 58 seconds) and from skin contact to inflation 3 minutes and 12 seconds (SD ± 42 seconds). There was a clinical significant increase in SBP and DBP after balloon inflation (Figure 1).

## Conclusion

REBOA could stop pelvic bleedings and improve central perfusion during massive bleeding. The technique appears feasible in the pre-hospital environment with an insertion time approximating 3 minutes after training. To treat the right patients at the right time requires additional decision-making skills for pre-hospital crews.

## Conflict of interest

The authors state no conflicts of interest.

